# Insights into the Dynamic Electron–Hole Separation Process Induced by a Trapped Electron in Lead Halide Perovskites in the Presence of Solutions

**DOI:** 10.1021/jacsau.4c01261

**Published:** 2025-03-18

**Authors:** Yunxuan Ding, Yujie Shen, Ming-Hsien Lee, Haifeng Wang, P. Hu, Meilan Huang

**Affiliations:** † School of Chemistry and Chemical Engineering, The Queen’s University of Belfast, Belfast BT9 5AG, U.K.; ‡ Department of Physics, Tamkang Univeristy, New Taipei, 25137, Taiwan; § Key Laboratory for Advanced Materials, Centre for Computational Chemistry and Research Institute of Industrial Catalysis, East China University of Science and Technology, Shanghai 200237, China; ∥ School of Physical Science and Technology, ShanghaiTech University, Shanghai 201210, China

**Keywords:** perovskite solar cells, electron localization, ab initio molecular dynamics, electron−hole separation, density functional theory

## Abstract

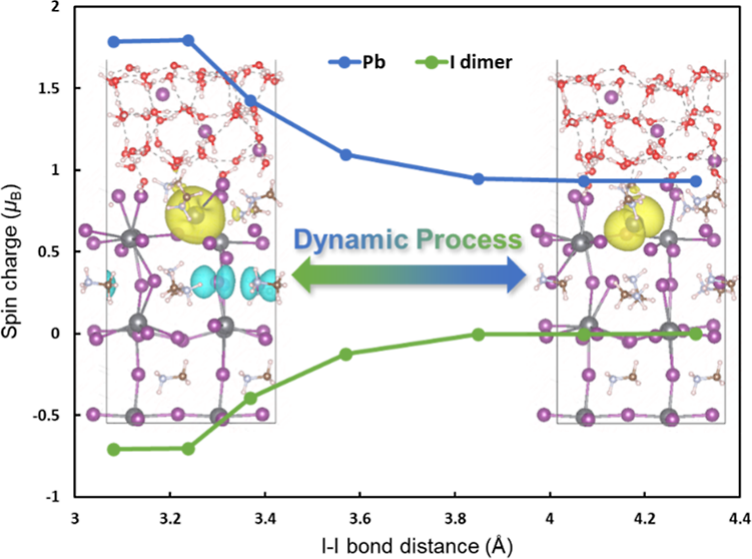

Metal halide perovskite solar cells show great promise, in terms of their high-power conversion efficiency. However, the dynamic electron–hole separation process remains elusive. Using ab initio molecular dynamics, we discover that the presence of photogenerated electron trapped at a Pb^2+^ ion can induce significant electron–hole separations on the CH_3_NH_3_PbI_3_ perovskite in the presence of HI solution. In this dynamic process, the separated electron is transferred to the Pb^+^ ion to form a Pb^0^ atom, while the separated hole is trapped in an I dimer. The reason behind this induced electron–hole separation is clearly revealed. Furthermore, the charge carrier transfer mechanism is elucidated, which not only explains the carrier migration but also the degradation of the perovskite in a humid environment. Comparing the atomic motions in CH_3_NH_3_PbI_3_ and CH_3_NH_3_PbCl_3_ quantitatively demonstrates that CH_3_NH_3_PbI_3_ is more active but less stable than CH_3_NH_3_PbCl_3_. The proposed mechanism for the electron–hole separation mechanism and perovskite degradation in humid conditions provides insights into the design of a highly efficient perovskite with good stability.

## Introduction

Hybrid organic–inorganic halide perovskite solar cells (PSCs) have been rapidly developed since the first invention in 2009,^1^ with its power conversion efficiency increasing from 3.8% to 26.7%.^2^ As promising next-generation photovoltaic materials, the PSCs not only have excellent properties, including the suitable band gap,^3^ long charge carrier diffusion length,^4^ high photoabsorption coefficient,^5^ and long carrier lifetime,^6^ along with commercial potential that ensures the economic feasibility.^7^ These extraordinary advantages prompt the PSCs to be widely employed in solar cells, light-emitting diodes,^8^ photodetectors,^9^ and lasers.^10^

Despite the high performance of the PSCs, the factors determining photovoltaic efficiency remain elusive. It is widely acknowledged that retarding nonradiative electron–hole recombination rates constitutes an efficient strategy for facilitating charge carrier transfer.^11^ To separate the electron–hole pair, the presence of trap states for both electrons and holes is of significance,^12−14^ as evidenced by the critical role of carrier localization within PSCs. Consequently, the investigation of charge carrier-trapped states has drawn significant attention over the past decade.^15−19^

It is noteworthy that intrinsic defects are inherently introduced during the synthesis of PSCs and have been posited as pivotal factors in carrier trapping and electron–hole pair recombination.^20^ As a result, extensive investigations have centered on vacancies of iodine(I) and lead (Pb) in the CH_3_NH_3_PbI_3_ (MAPbI_3_) perovskite. For instance, Long and co-workers observed a Pb vacancy in the perovskite that results in a shallow hole trap closing to the valence band, which facilitates hole transport and concurrently delay electron–hole pair recombination.^21^ Angelis, Petrozza, and co-workers identified that intrinsic charge traps were caused by the peculiar iodine redox chemistry. They suggested that the high photovoltaic efficiency and long-term stability of solar cells can be achieved by adjusting the defect redox chemistry.^22,23^ Our group made a pertinent observation, elucidating that photogenerated electrons exhibit delocalization on the defect-free MAPbI_3_ surface but can be readily localized on perovskites containing I vacancies.^24^ Furthermore, Zhang and Sit reported the presence of excess electrons trapped in both the shallow-level and deep-level states on the MAPbI_3_ surface containing I vacancies. Intriguingly, these vacancies, while initially more stable on the surface, migrate from the top layer to the sublayer by trapping photogenerated electrons, leading to the formation of deep trap states that accelerate undesired photodegradation.^25^ Collectively, the previous works underscore the dual nature of defects: shallow trap states caused by some defects facilitate the photogenerated charge carrier trap and transport, whereas deep trap states caused by other defects accelerate electron–hole pair recombination and structural degradation. It is evident that defects exert a profound influence not only on the photovoltaic efficiency but also on the overall stability of the perovskite material.

While the presence of defects in perovskite materials is inevitable, an excess of such defects readily causes deep trap states, which can detrimentally affect photogenerated carrier migration, leading to perovskite degradation. As a solution, our previous work has unveiled a promising avenue for an alternative form of carrier localization: We have identified the presence of a hole-trapped dimer state within the defect-free MAPbI_3_ bulk material and elucidated the mechanism of hole diffusion, characterized by a low energy barrier.^26^ In addition to the photogenerated hole localized in bulk, we also observed a shallow trap state of the charge carrier on the defect-free MAPbI_3_ surface with different terminations in the presence of the solution.^27^ Furthermore, as a potential remedy, numerous experimental studies have indicated that the defect passivation may reduce the concentration of the surface defects, consequently enhancing both the efficiency and stability of the perovskites.^28−32^ These findings emphasize the influence of the surrounding environment, suggesting that solvation and ligand effects are critical factors in the study of charge carrier-trapped states. Although significant progress has been achieved in comprehending trap states and photogenerated carriers, most reported carrier-transport mechanisms have relied on static quantum chemical calculations and band structure analysis, with little exploration of the dynamic structural transformations of perovskites. Additionally, intrinsic mechanisms governing electron–hole pair separation and the migration of photogenerated carriers remain elusive.

In this work, a first-principles study was conducted to elucidate the localization of photogenerated carriers in the MAI-terminated MAPbI_3_(001) perovskite surface, the most stable surface in the MAPbI_3_ perovskite.^27^ It was observed that a localized electron on Pb^2+^ can surprisingly induce electron–hole separations, leading to a coexistence state featuring both trapped electron and trapped hole on the MAPbI_3_ surface in the presence of the solution, as revealed through the ab initio molecular dynamics (AIMD) simulations. After meticulously examining its dynamic structural transformations, we identified a distinctive hole-trapped state associated with an I dimer. Furthermore, a mechanism for perovskite degradation in a humid environment—an important issue of perovskite materials—was proposed, aiming to provide insights into the stability and performance of perovskite materials.

## Results and Discussion

**Figure 1:**
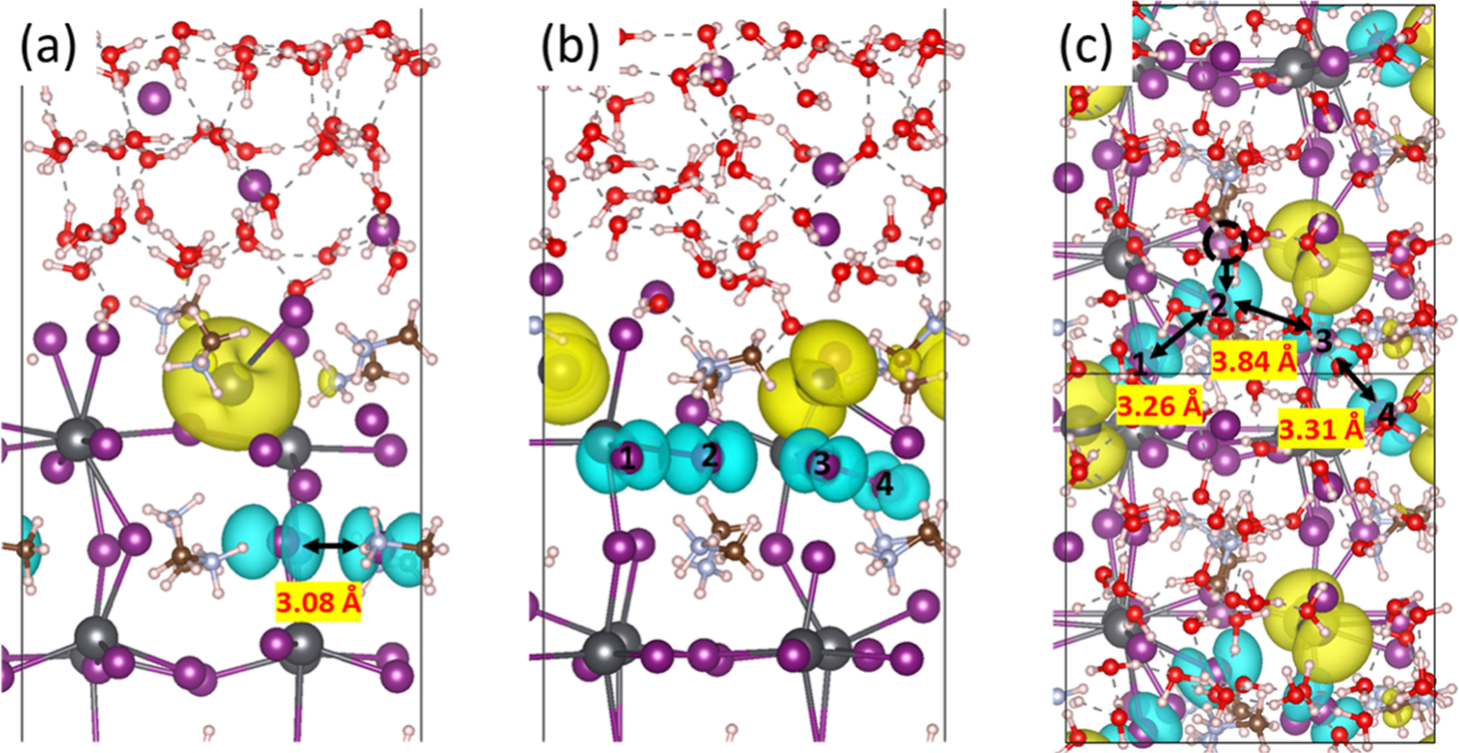
Snapshots with spin densities from MD simulation of MAI-terminated MAPbI_3_ surfaces in the presence of 3.15 mol/L HI solutions with an extra electron. Side view of (a) one I dimer structure, (b) side, and (c) top views of two I dimer structure, respectively. The iso-value of spin densities is 0.002 e/Å^3^. The spin-up and spin-down charge densities are represented by yellow and cyan, respectively. Black: Pb; purple: I; white: H; brown: C; silver: N; red: O. This color scheme is used throughout the paper. The characteristic distance is marked by the double-arrow line.

To simulate the photoexcited structure of perovskite, an additional electron was introduced into the MAPbI_3_ system. The AIMD calculations were carried out at room temperature to investigate the dynamic structural transformations of the MAPbI_3_ surface when exposed to a HI-saturated solution at a concentration of 3.15 mol/L, closely mirroring the real experimental situation (3.162 mol/L).^33^ In this system, a localized electron on Pb^2+^ was found, resulting in the formation of Pb^+^. Additionally, an unexpected hole-trapped state was detected during the AIMD simulation. The hole localized on an I dimer with an I–I distance of 3.08 Å (Figure 1a), while the electron remained trapped on the Pb ion, establishing the coexistence of both electron- and hole-trapped states. The AIMD trajectory provides insights into the dynamic formation of the I dimer, which occurred through a series of sequential steps: First, the Pb–I bond weakened after the electron trapped on the Pb, leading to the liberation of a weakly bonded I^–^. Subsequently, weakly bonded I^–^ migrated to the sublayer of the perovskite. Finally, this I^–^ interacted with another I^–^ to form an I dimer, resulting in hole trapping.

It is well established that electron–hole pair recombination can occur readily; however, carrier transport can significantly impede this recombination process. To assess the stability of this coexistent electron–hole structure, a prolonged AIMD simulation was conducted. The MD simulation revealed that this coexisting trap state of the electron and hole was frequently observed within the dynamic simulations at room temperature. Notably, another I dimer structure emerged (Figure 1b,c) and disappeared cyclically during the MD simulations. The weakly bonded I^–^ not only had the capacity to migrate to the sublayer but could also move to a neighboring I^–^ on the top layer, resulting in the formation of another distinct type of I dimer. With the formation of this I dimer, the hole was trapped within the dimeric structure. Concurrently, surplus electrons were released from the I dimer, along with the separation of the electron–hole pair. To discern the distribution of spin charge within the system, a spin charge density analysis was employed. It was observed that the total spin charge density of two iodides changed from −2 to −1.5 |e| during the formation of the I dimer, indicating the release of approximately 0.5 electron from the I dimer. These released electrons could either be trapped on the Pb^+^ (Figure 1a) or on another coordination-saturated Pb^2+^ (Figure 1b,c). Importantly, electrons trapped on another Pb^2+^ promoted the breaking of the nearby Pb–I bond, thereby further expediting the separation of the electron–hole pair and subsequently generating additional I dimers. This additional formation of an I dimer released another 0.5 electron, which were in turn trapped on the Pb^2+^, ultimately leading to the formation of another Pb^+^ (Figure 1b,c). This discovery holds potential significance in elucidating the pathways of photogenerated charge carrier transfer and the degradation mechanisms of perovskite materials in humid conditions.

**Figure 2:**
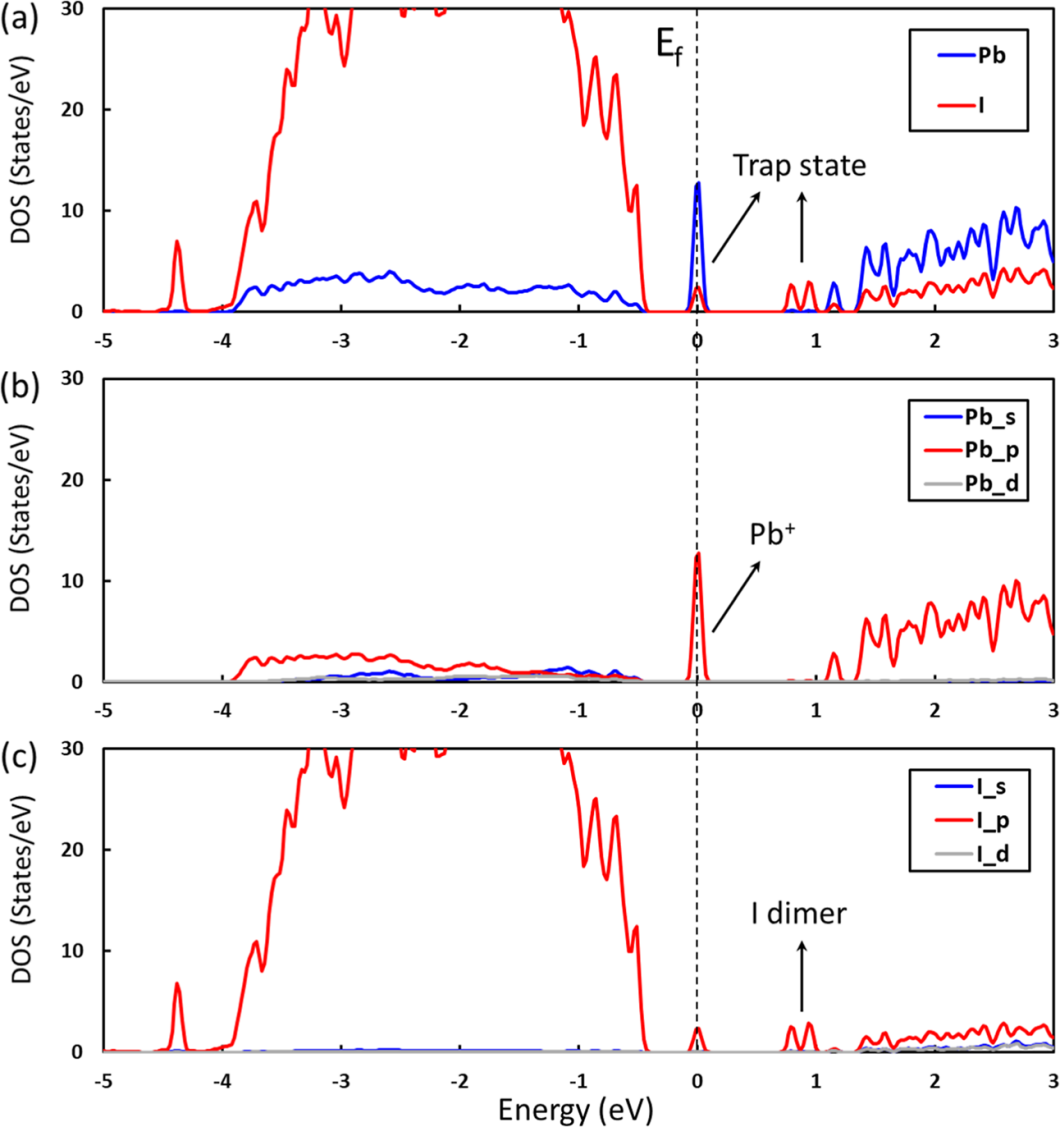
(a) TDOS of the electron–hole coexisting state of Pb and I on the MAPbI_3_ surface with SOC. PDOS of (b) Pb and (c) I in the electron–hole coexisting system with SOC. The Fermi level is set at 0 eV.

To characterize the electronic structure of the system, calculations of the density of states (DOS) were performed for the electron–hole coexisting state on the MAPbI_3_ surface, incorporating spin–orbit coupling (SOC) (Figure 2). The total DOS calculations show that there are two polaron peaks in the band gap (Figure 2a), localized on Pb and I, respectively. The projected DOS (PDOS) analysis of Pb and I demonstrates that these polaron peaks are mainly composed of the 6p orbitals of Pb and the 5p orbitals of I (Figure 2b,c). Furthermore, a detailed DOS analysis of these polaron peaks unravels that the occupied polaron peak of Pb, located 0.41 eV above the valence band maximum (VBM), corresponded to the Pb^+^ with trapped electrons (Figure S1a). Meanwhile, the unoccupied polaron peaks below the conduction band minimum (CBM) were assigned to be the I dimer structure (Figure S1b). To gain a comprehensive understanding of the advantages associated with the coexistent electron–hole state, a wave function analysis in the real space was carried out. As depicted in Figure S2, the CBM was found to be delocalized on Pb atoms, whereas the Pb^+^ with trapped electrons appears to be a surface state. This configuration can facilitate the transfer of photogenerated electrons. Concurrently, the delocalized VBM on the I atoms served as a favorable hole transport channel (Figure S3). Moreover, the polaron peak at −4.38 eV, belonging to the 5p orbital of I, clearly reveals bonding between two I atoms (Figure S3b), providing further evidence of I dimer formation. This dimer configuration could act as a supporter of ion migration, thereby enhancing hole transfer. To contrast the effect of the I dimer structure, the DOS of the perovskite with electrons localized on Pb was also analyzed/compared to that without the formation of I dimer. In this case, the trap states associated with the I dimer structure above the Fermi level disappear, while the surface states of Pb^+^ remain. These findings indicate the crucial role of the I dimer as a hole trapper, influencing the recombination/separation of electron–hole pairs.

**Figure 3:**
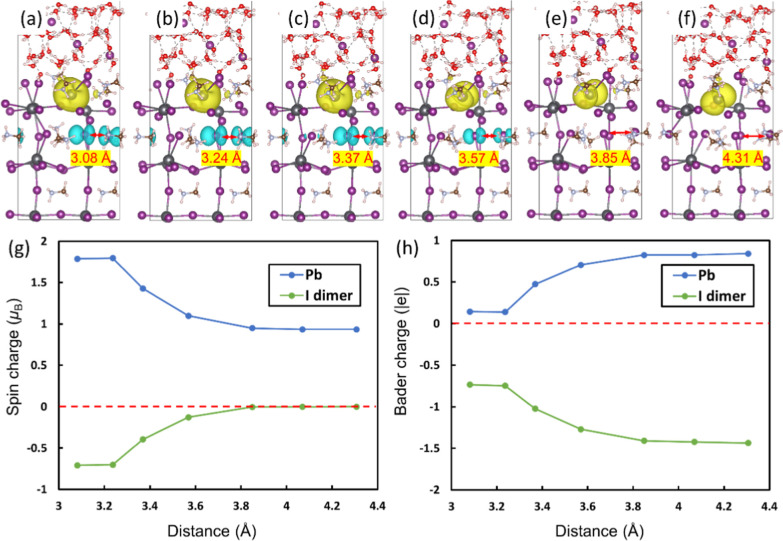
Geometric structures and spin densities of (a–f) series of MAI-terminated MAPbI_3_ surfaces in the presence of 3.15 mol/L HI solution with an extra electron from AIMD simulations. The iso-value is 0.002 e/Å^3^. (g) Spin densities of the electron trapped at Pb and the hole trapped at the I dimer and (h) Bader charges of the valence states of Pb and I dimer as a function of the I dimer distance.

A previous study reported that the typical distance of I dimer was approximately 3.30 Å, whereas the equilibrium distance between two iodide ions in pristine MAPbI_3_ was 4.4 Å.^26^ Thus, to explore the transformation mechanism of the I dimer, a series of MAPbI_3_ structures from the MD simulations were investigated, encompassing various I dimer structures with bond distances ranging from 3.0 to 4.4 Å (Figure 3a–f). Interestingly, the spin density of the I dimer remains constant at −0.71 μ_B_ when the bond distance is less than 3.24 Å, aligned with the optimized bond distance of the I dimer. As the bond distance of the I dimer increases, its spin density gradually approaches 0 μ_*B*_ until the I dimer dissociates into two separate I^–^ ions at a distance of 3.85 Å (Figure 3g). Furthermore, it is noteworthy that the spin density of the electron trapped on Pb exhibits a similar change trend to that of the hole trapped on the I dimer. This observation implies that the surplus electrons resulting from the separation of the electron–hole pair are predominantly localized on the Pb^+^.

To further identify the oxidation state of the Pb and I dimers and to understand the electron-transfer dynamics, Bader charge analysis was conducted (Figure 3h). With an additional electron trapped on Pb^2+^ in this system, it was observed that Pb^2+^ is reduced to Pb^+^ first. During the formation of the I dimer, two I^–^ ions combine, subsequently releasing some electrons. These released electrons are then accepted by Pb^+^, further reducing Pb^+^ and bringing the oxidation state of Pb close to ∼0. This phenomenon offers a plausible explanation for the commonly observed Pb^0^ state in experiments involving PSCs.^34,35^ The separation of the electron–hole pair occurs concomitantly with the formation of the I dimer, but the recombination of the electron–hole pair happens when the I dimer transforms into two separate I^–^ ions. Moreover, the energy plot shows that as the I–I bond distance increases, the separated electron and hole recombine, leading to a decrease in energy (Figure S4), which aligns well with the experimental observation of favored electron–hole pair recombination. During the AIMD simulation, an intriguing observation was made as the bond distance of the I dimer was extended: one of the iodides in the I dimer approaches the third iodide in the same layer, leading to the formation of a new I dimer as the original I dimer disappears (Figure S5). Meanwhile, the trapped hole migrates from the former I dimer to the newly formed I dimer, establishing a chain for transporting charge carriers (Figure S5). These structural alterations elucidate a potential mechanism for the migration of hole trap states (Video S1).

Having identified the coexisting trap states of an electron and a hole in the MD simulations, we are in the position to investigate whether this state is a stable state. Namely, can it persist after structural optimization? To address this inquiry, an extended supercell (*p*(4 × 2) supercell) with coexisting electron and hole states from the MD simulation was optimized with an additional introduced electron (see details in the Supporting Information). The result reveals the stable coexistence of two Pb^+^ ions and two I dimers (Figure S6), confirming the persistence of this coexisting state in both dynamic and optimized calculations. Obviously, the introduced electron is trapped on one Pb^2+^, while the separated electron and hole are localized on another Pb^2+^ and two I dimers, respectively. Furthermore, the optimized bond distances of these two I dimers measure 3.29 and 3.33 Å, respectively, which agree well with the typical I dimer distances in the previous work.^26^

**Figure 4:**
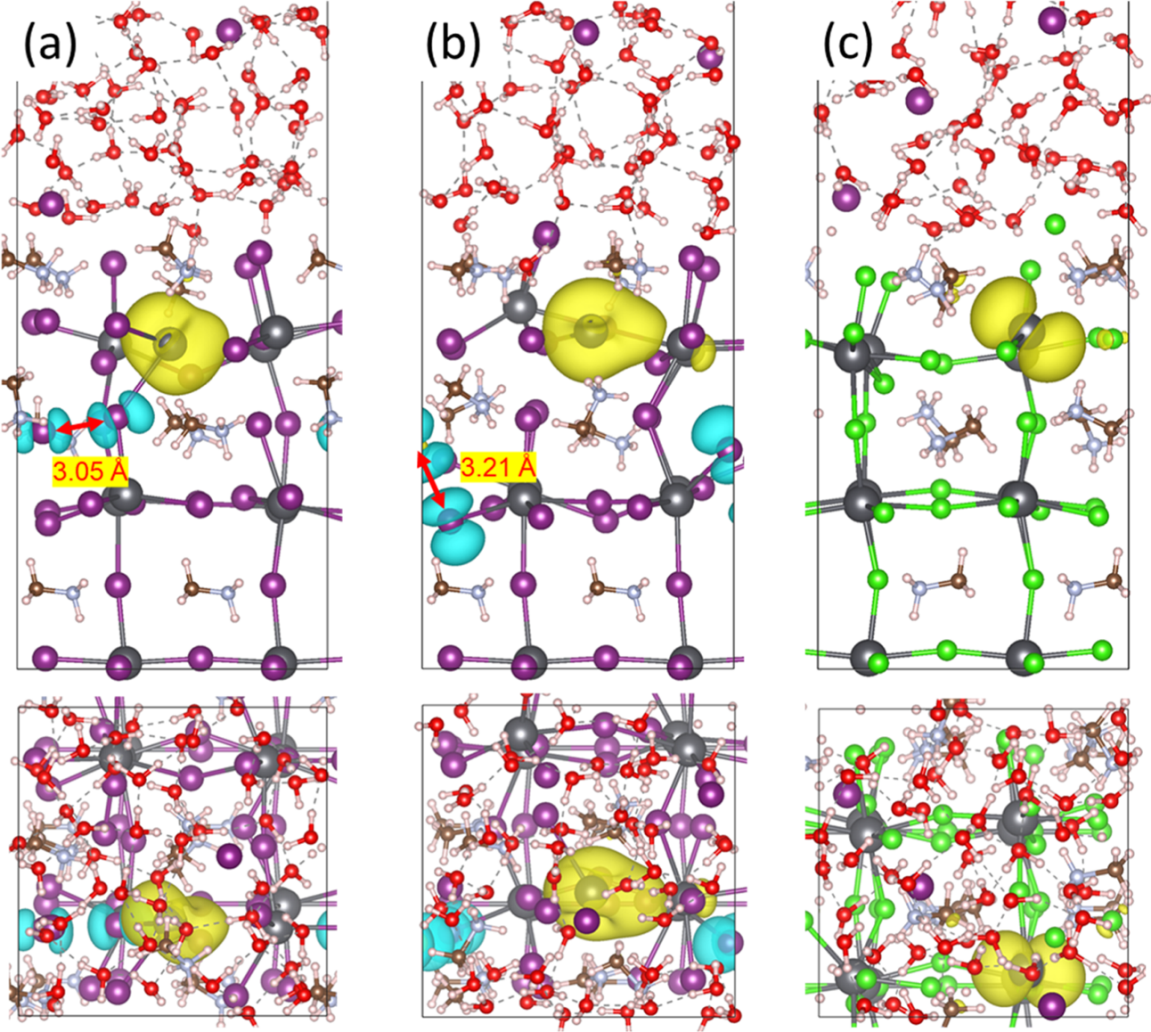
Side views (upper) and top views (lower) of geometric structures and spin densities of MAPbI_3_ surface in the presence of HI solutions of 1.05 mol/L (a) and 2.10 mol/L (b), and MAPbCl_3_ surface in the presence of 3.15 mol/L HI solution (c). The iso-value is 0.002 e/Å^3^. Green: Cl.

It is well known that halide perovskites are prone to instability when exposed to a humid environment,^36^ although the moisture has been suggested to increase the lifetime of charge carriers.^37^ Thus, the different concentrations of the HI solutions are then taken into consideration in this work. To explore the impact of different concentrations of HI solutions on MAPbI_3_, HI solutions of 1.05 and 2.10 mol/L were added above the surfaces (Figure 4a,b). The MD simulations show the coexistence of the hole trapped on the I dimer and the electron trapped on a Pb ion under both solution conditions, mirroring the behavior observed for MAPbI_3_ in an HI-saturated (3.15 mol/L) solution (Figure 1a). Furthermore, a water-only environment and dry condition were used to explore the role of the water solvent in dynamic electron–hole separation. It was found that the electron–hole separation readily occur in the presence of water molecules. However, under dry conditions, the additional electron delocalized across the entire system (Figure S7), and no electron–hole separation was observed throughout the MD simulation. This suggests that the presence of water molecules is critical, while HI may not be essential for the dynamic electron–hole separation/recombination. Specifically, solvation indirectly expedites the formation of the I dimer. Concomitant with structural transformations, the hole and electron are trapped on the I dimer and coordination-unsaturated Pb, respectively, which would further weaken the Pb–I bond, contributing to perovskite degradation.

For comparison, a parallel investigation involving another halide perovskite, MAPbCl_3_, was conducted in which identical conditions and methodology were applied. Over the same time scale, no evidence of a Cl dimer formation was observed (Figure 4c). In pursuit of understanding the underlying cause, the canonically averaged standard deviation^38^ was used, in which the positions of I and Cl atoms were calculated according to the following formula
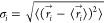
1where  represents the location of atom *i* at time t, and the angular bracket indicates ensemble and time averaging along the last 4 ps MD trajectories. As shown in Table 1, the standard deviation of Cl atoms is 0.302 Å in the presence of HI solution of 3.15 mol L^–1^, while those of I atoms in the presence of HI solutions of 3.15, 2.10, and 1.05 mol L^–1^ are 0.405, 0.359, and 0.387 Å, respectively. These results unequivocally demonstrate that the migration distance of Cl is significantly smaller than those of I, indicating the inherent challenge in the formation of Cl dimers. We suggest that the different redox chemistries of I and Cl may offer an explanation for the difference in dimer formation of I and Cl. The stronger oxidizing ability of Cl, relative to I, culminates in the stronger ability to gain electrons for Cl compared to I. Consequently, the release of electrons to form the I dimer becomes more feasible. Furthermore, the larger standard deviation of I/Cl atomic motions corresponds to a more rapid degradation of the metal halide perovskites. This disparity enhances the mobility of the photogenerated carriers but simultaneously undermines the stability of the perovskite. This may provide a rationale behind the better photovoltaic efficiency observed in MAPbI_3_ compared to MAPbCl_3_.

**Table 1:** Standard Deviations in the Positions of I and Cl Atoms in MAPbI_3_ and MAPbCl_3_ under Different Concentrations of HI Solutions

conditions	standard deviations (Å)
MAPbI_3_ (3.15 mol/L)	0.405
MAPbI_3_ (2.10 mol/L)	0.359
MAPbI_3_ (1.05 mol/L)	0.387
MAPbCl_3_ (3.15 mol/L)	0.302

The reliability of the theoretical calculations can be supported by several experimental techniques. For instance, the localization of the electrons on a Pb^2+^ ion leads to its reduction to Pb^+^ or Pb^0^, a phenomenon confirmed by high-resolution X-ray photoelectron spectroscopy, which defected the presence of Pb^0^ in the halide perovskite.^35^ More specifically, the valence state, coordination number, and local atomic environment of Pb can be precisely analyzed using an X-ray absorption fine structure. In the case of I dimer formation, Baran and co-workers employed absorption spectroscopy to a distinct absorption peak at around 500 nm, characteristic of I dimers. The quantification of these dimers was achieved by applying the Beer–Lambert Law to determine their molar extinction coefficients.^39^ Additionally, transient fluorescence spectroscopy and transient absorption spectroscopy can measure the lifetimes of excitons in different perovskites such as revealing the differences between MAPbI_3_ and MAPbCl_3_. Furthermore, grazing incidence X-ray diffraction has provided evidence of partial decomposition and photodegradation in the halide perovskite.^34^

## Conclusions

In summary, by performing AIMD simulations coupled with meticulous charge analyses, we unveiled for the first time that a localized electron on Pb induces cyclically a dynamic separation/recombination of electron–hole pairs, leading to the consequent reduction of Pb^+^ or other Pb^2+^ ions. This, in turn, results in the coexistence of trapped electrons and trapped holes within the MAPbI_3_ perovskite. We revealed that once the electron is localized on Pb^2+^, the Pb–I bond is substantially weakened. Subsequently, the weakly bonded iodide migrates to a nearby iodide, leading to the formation of an I dimer. This process concurrently causes the release of certain electrons from the dimer, thereby promoting the separation of electron–hole pairs. The dynamic structural transformation of the MAPbI_3_ perovskite was visualized and related to the charge carrier transfer mechanism. This charge carrier transfer mechanism serves as a comprehensive explanation not only for charge carrier migration but also for observed perovskite degradation in humid environments. Moreover, the difficult formation of Cl dimer in the MAPbCl_3_ perovskite was demonstrated, indicating a poor mobility of the photogenerated carriers, which accounts for the comparatively inferior photovoltaic efficiency of MAPbCl_3_ compared with MAPbI_3_.

## Methods

All the density functional theory (DFT) calculations in this work were performed using the Vienna ab initio simulation program (VASP).^40,41^ The projector-augmented wave method^41,42^ was utilized to evaluate the interactions between the valence electrons and ions, and the cutoff energy of plane-wave basis expansion was set to be 400 eV with 4 × 4 × 1 Monkhorst–Pack *k*-point mesh sampling for Brillouin-zone integration for the *p*(2 × 2) surface unit cell. The valence electrons of 5d^10^6s^2^6p^2^ for the lead (Pb) atom, 5s^2^5p^5^ for the iodine(I) atom, 3s^2^3p^5^ for the chlorine (Cl) atom, 2s^2^2p^2^ for the carbon (C) atom, 2s^2^2p^3^ for the nitrogen (N) atom, 2s^2^2p^4^ for the oxygen (O) atom, 1s^1^ for the hydrogen (H) atom, and 3s^2^3p^6^4s^1^ for the potassium (K) atom were chosen. The generalized gradient approximation was used with the Perdew–Burke–Ernzerhof functional (PBE)^43^ to perform all spin-polarized calculations. The optimized structures were reached when the force on the relaxed atoms was less than 0.05 eV/Å. For MAPbI_3_, our previous work showed that the DFT + *U* method can yield similar structures and reasonable energies as PBE0 and HSE06 methods, where the Hubbard-type correction was set on Pb 6p and I 5p orbitals with effective U values of 9 and 8 eV, respectively.^24,27^ In this work, the same approach, namely, DFT + *U* was utilized for structure optimizations and the AIMD simulations. For the van der Waal correction, the DFT-D3 method was employed to describe the weak interaction in the system.^44,45^ Dipole corrections were applied along the surface’s normal direction. To simulate photogenerated electrons, an extra electron was added in the system, in which similar approaches have been verified in previous works.^24,46−48^ The AIMD calculations were carried out to simulate all of the systems in the presence of solutions at a constant temperature (300 K) with a time step of 1 fs and a total time over 10 ps. To evaluate a more reliable DOS analysis, the SOC^49^ and HSE06 hybrid functional^50^ were introduced in this work (Figure S8).

The cubic phase of MAPbI_3_ was chosen with the calculated lattice constant of 6.29 Å, which agrees well with the X-ray diffraction experimental data of ∼6.30 Å.^51−53^ A *p*(2 × 2) supercell stoichiometric MAI-terminated MAPbI_3_(001) surface with three atomic layers was constructed, while the bottom layer was fixed and the others relaxed. To simulate the slabs in the presence of HI solutions with different concentrations, *a* ∼ 10 Å solution layer composed of 52 H_2_O molecules and one/two/three HI molecules were placed above the surfaces.^27^ The concentrations of the solution containing one, two, and three HI molecules are 1.05, 2.10, and 3.15 mol/L, respectively, the highest of which is consistent with the experiment condition (3.162 mol/L).^33^ A 10 Å vacuum layer was positioned at the upper boundary of the solution to effectively eliminate periodic boundary effects along the *Z*-axis.

## Supporting Information Available

Electronic property of the MAPbI_3_ with a I dimer, energy plot of the separated electron and hole recombination, iodide migration in the I dimer, coexisting state of the trapped electron and hole in the optimized structure, delocalized electron on MAPbI_3_ in dry conditions, and comparison of DOS calculated by different functionals (PDF)
Video of structural alterations (MP4)

## References

[ref1] Kojima A.; Teshima K.; Shirai Y.; Miyasaka T. Organometal halide perovskites as visible-light sensitizers for photovoltaic cells. J. Am. Chem. Soc. 2009, 131, 6050–6051. 10.1021/ja809598r. 19366264

[ref2] Best Research-Cell Effciencies Chart. https://www.nrel.gov/pv/cell-efficiency.html. (Accessed December 3, 2024).

[ref3] Liao W.; Zhao D.; Yu Y.; Shrestha N.; Ghimire K.; Grice C. R.; Wang C.; Xiao Y.; Cimaroli A. J.; Ellingson R. J.; Podraza N. J.; Zhu K.; Xiong R. G.; Yan Y. Fabrication of efficient low-bandgap perovskite solar cells by combining formamidinium tin iodide with methylammonium lead iodide. J. Am. Chem. Soc. 2016, 138, 12360–12363. 10.1021/jacs.6b08337. 27622903

[ref4] Zhumekenov A. A.; Saidaminov M. I.; Haque M. A.; Alarousu E.; Sarmah S. P.; Murali B.; Dursun I.; Miao X.-H.; Abdelhady A. L.; Wu T.; Mohammed O. F.; Bakr O. M. Formamidinium lead halide perovskite crystals with unprecedented long carrier dynamics and diffusion length. ACS Energy Lett. 2016, 1, 32–37. 10.1021/acsenergylett.6b00002.

[ref5] Pazos-Outon L. M.; Szumilo M.; Lamboll R.; Richter J. M.; Crespo-Quesada M.; Abdi-Jalebi M.; Beeson H. J.; Vrucinic M.; Alsari M.; Snaith H. J.; Ehrler B.; Friend R. H.; Deschler F. Photon recycling in lead iodide perovskite solar cells. Science 2016, 351, 1430–1433. 10.1126/science.aaf1168. 27013728

[ref6] Peng W.; Miao X.; Adinolfi V.; Alarousu E.; El Tall O.; Emwas A. H.; Zhao C.; Walters G.; Liu J.; Ouellette O.; Pan J.; Murali B.; Sargent E. H.; Mohammed O. F.; Bakr O. M. Engineering of CH_3_NH_3_PbI_3_ perovskite crystals by alloying large organic cations for enhanced thermal stability and transport properties. Angew. Chem., Int. Ed. 2016, 55, 10686–10690. 10.1002/anie.201604880. 27468159

[ref7] Yao Z.; Zhang F.; Guo Y.; Wu H.; He L.; Liu Z.; Cai B.; Guo Y.; Brett C. J.; Li Y.; Srambickal C. V.; Yang X.; Chen G.; Widengren J.; Liu D.; Gardner J. M.; Kloo L.; Sun L. Conformational and compositional tuning of phenanthrocarbazole-based dopant-free hole-transport polymers boosting the performance of perovskite solar cells. J. Am. Chem. Soc. 2020, 142, 17681–17692. 10.1021/jacs.0c08352. 32924464 PMC7584363

[ref8] Xiong W.; Tang W.; Zhang G.; Yang Y.; Fan Y.; Zhou K.; Zou C.; Zhao B.; Di D. Controllable p- and n-type behaviours in emissive perovskite semiconductors. Nature 2024, 633, 344–350. 10.1038/s41586-024-07792-4. 39261614

[ref9] Dou L.; Yang Y. M.; You J.; Hong Z.; Chang W. H.; Li G.; Yang Y. Solution-processed hybrid perovskite photodetectors with high detectivity. Nat. Commun. 2014, 5, 5404 10.1038/ncomms6404. 25410021

[ref10] Xing G.; Mathews N.; Lim S. S.; Yantara N.; Liu X.; Sabba D.; Gratzel M.; Mhaisalkar S.; Sum T. C. Low-temperature solution-processed wavelength-tunable perovskites for lasing. Nat. Mater. 2014, 13, 476–480. 10.1038/nmat3911. 24633346

[ref11] Long R.; Liu J.; Prezhdo O. V. Unravelling the effects of grain boundary and chemical doping on electron-hole recombination in CH_3_NH_3_PbI_3_ perovskite by time-domain atomistic simulation. J. Am. Chem. Soc. 2016, 138, 3884–3890. 10.1021/jacs.6b00645. 26930494

[ref12] Yuan H.; Sun H.; Shi Y.; Wang J.; Bian A.; Hu Y.; Guo F.; Shi W.; Du X.; Kang Z. Cooperation of carbon doping and carbon loading boosts photocatalytic activity by the optimum photo-induced electron trapping and interfacial charge transfer. Chem. Eng. J. 2023, 472, 144654 10.1016/j.cej.2023.144654.

[ref13] Caselli V. M.; Thieme J.; Jöbsis H. J.; Phadke S. A.; Zhao J.; Hutter E. M.; Savenije T. J. Traps in the spotlight: How traps affect the charge carrier dynamics in Cs_2_AgBiBr_6_ perovskite. Cell Rep. Phys. Sci. 2022, 3, 101055 10.1016/j.xcrp.2022.101055.

[ref14] Li Y.; Jia Z.; Yang Y.; Yao F.; Liu Y.; Lin Q. Shallow traps-induced ultra-long lifetime of metal halide perovskites probed with light-biased time-resolved microwave conductivity. Appl. Phys. Rev. 2023, 10, 011406 10.1063/5.0129883.

[ref15] Agiorgousis M. L.; Sun Y. Y.; Zeng H.; Zhang S. Strong covalency-induced recombination centers in perovskite solar cell material CH_3_NH_3_PbI_3_. J. Am. Chem. Soc. 2014, 136, 14570–14575. 10.1021/ja5079305. 25243595

[ref16] Wang J.; Li W.; Yin W. J. Passivating detrimental DX centers in CH_3_NH_3_PbI_3_ for reducing nonradiative recombination and elongating carrier lifetime. Adv. Mater. 2020, 32, 1906115 10.1002/adma.201906115. 31840331

[ref17] Ahn N.; Kwak K.; Jang M. S.; Yoon H.; Lee B. Y.; Lee J. K.; Pikhitsa P. V.; Byun J.; Choi M. Trapped charge-driven degradation of perovskite solar cells. Nat. Commun. 2016, 7, 13422 10.1038/ncomms13422. 27830709 PMC5110646

[ref18] Ni Z.; Bao C.; Liu Y.; Jiang Q.; Wu W. Q.; Chen S.; Dai X.; Chen B.; Hartweg B.; Yu Z.; Holman Z.; Huang J. Resolving spatial and energetic distributions of trap states in metal halide perovskite solar cells. Science 2020, 367, 1352–1358. 10.1126/science.aba0893. 32193323

[ref19] Yao Q.; Li H.; Xue J.; Jiang S.; Zhang Q.; Bao J. Promoting photocatalytic h_2_ evolution through retarded charge trapping and recombination by continuously distributed defects in methylammonium lead iodide perovskite. Angew. Chem., Int. Ed. 2023, 62, e202308140 10.1002/anie.202308140. 37395373

[ref20] Xing G.; Mathews N.; Sun S.; Lim S. S.; Lam Y. M.; Gratzel M.; Mhaisalkar S.; Sum T. C. Long-range balanced electron- and hole-transport lengths in organic-inorganic CH_3_NH_3_PbI_3_. Science 2013, 342, 344–347. 10.1126/science.1243167. 24136965

[ref21] Qiao L.; Fang W. H.; Long R. The interplay between lead vacancy and water rationalizes the puzzle of charge carrier lifetimes in CH_3_NH_3_PbI_3_: Time-domain ab initio analysis. Angew. Chem., Int. Ed. 2020, 59, 13347 10.1002/anie.202004192. 32337808

[ref22] Meggiolaro D.; Motti S. G.; Mosconi E.; Barker A. J.; Ball J.; Andrea Riccardo Perini C.; Deschler F.; Petrozza A.; De Angelis F. Iodine chemistry determines the defect tolerance of lead-halide perovskites. Energy Environ. Sci. 2018, 11, 702–713. 10.1039/C8EE00124C.

[ref23] Motti S. G.; Meggiolaro D.; Martani S.; Sorrentino R.; Barker A. J.; De Angelis F.; Petrozza A. Defect activity in lead halide perovskites. Adv. Mater. 2019, 31, e1901183 10.1002/adma.201901183. 31423684

[ref24] Peng C.; Chen J.; Wang H.; Hu P. First-principles insight into the degradation mechanism of CH_3_NH_3_PbI_3_ perovskite: Light-induced defect formation and water dissociation. J. Phys. Chem. C 2018, 122, 27340–27349. 10.1021/acs.jpcc.8b07294.

[ref25] Zhang L.; Sit P. H. L. Ab initio study of the dynamics of electron trapping and detrapping processes in the CH_3_NH_3_PbI_3_ perovskite. J. Mater. Chem. A 2019, 7, 2135–2147. 10.1039/C8TA09512D.

[ref26] Peng C.; Wang J.; Wang H.; Hu P. Unique trapped dimer state of the photogenerated hole in hybrid orthorhombic CH_3_NH_3_PbI_3_ perovskite: Identification, origin, and implications. Nano Lett. 2017, 17, 7724–7730. 10.1021/acs.nanolett.7b03885. 29125776

[ref27] Ding Y.; Shen Y.; Peng C.; Huang M.; Hu P. Unraveling the photogenerated electron localization on the defect-free CH_3_NH_3_PbI_3_(001) surfaces: Understanding and implications from a first-principles study. J. Phys. Chem. Lett. 2020, 11, 8041–8047. 10.1021/acs.jpclett.0c02105. 32893641

[ref28] Wang R.; Xue J.; Wang K. L.; Wang Z. K.; Luo Y.; Fenning D.; Xu G.; Nuryyeva S.; Huang T.; Zhao Y.; Yang J. L.; Zhu J.; Wang M.; Tan S.; Yavuz I.; Houk K. N.; Yang Y. Constructive molecular configurations for surface-defect passivation of perovskite photovoltaics. Science 2019, 366, 1509–1513. 10.1126/science.aay9698. 31857483

[ref29] Jiang Q.; Zhao Y.; Zhang X.; Yang X.; Chen Y.; Chu Z.; Ye Q.; Li X.; Yin Z.; You J. Surface passivation of perovskite film for efficient solar cells. Nat. Photonics 2019, 13, 460–466. 10.1038/s41566-019-0398-2.

[ref30] Tan S.; Yavuz I.; Weber M. H.; Huang T.; Chen C.-H.; Wang R.; Wang H.-C.; Ko J. H.; Nuryyeva S.; Xue J.; Zhao Y.; Wei K.-H.; Lee J.-W.; Yang Y. Shallow iodine defects accelerate the degradation of α-phase formamidinium perovskite. Joule 2020, 4, 2426–2442. 10.1016/j.joule.2020.08.016.

[ref31] Kim H.; Yoo S. M.; Ding B.; Kanda H.; Shibayama N.; Syzgantseva M. A.; Tirani F. F.; Schouwink P.; Yun H. J.; Son B.; Ding Y.; Kim B. S.; Kim Y. Y.; Park J.; Syzgantseva O. A.; Jeon N. J.; Dyson P. J.; Nazeeruddin M. K. Shallow-level defect passivation by 6H perovskite polytype for highly efficient and stable perovskite solar cells. Nat. Commun. 2024, 15, 5632 10.1038/s41467-024-50016-6. 38965276 PMC11224362

[ref32] Qu Z.; Zhao Y.; Ma F.; Mei L.; Chen X. K.; Zhou H.; Chu X.; Yang Y.; Jiang Q.; Zhang X.; You J. Enhanced charge carrier transport and defects mitigation of passivation layer for efficient perovskite solar cells. Nat. Commun. 2024, 15, 8620 10.1038/s41467-024-52925-y. 39366950 PMC11452620

[ref33] Park S.; Chang W. J.; Lee C. W.; Park S.; Ahn H.-Y.; Nam K. T. Photocatalytic hydrogen generation from hydriodic acid using methylammonium lead iodide in dynamic equilibrium with aqueous solution. Nat. Energy 2016, 2, 16185 10.1038/nenergy.2016.185.

[ref34] Lu L.; Shen K. C.; Wang J.; Su Z.; Li Y.; Chen L.; Luo Y.; Song F.; Gao X.; Tang J. X. Interaction of the cation and vacancy in hybrid perovskites induced by light illumination. ACS Appl. Mater. Interfaces 2020, 12, 42369–42377. 10.1021/acsami.0c11696. 32840343

[ref35] Liang J.; Hu X.; Wang C.; Liang C.; Chen C.; Xiao M.; Li J.; Tao C.; Xing G.; Yu R.; Ke W.; Fang G. Origins and influences of metallic lead in perovskite solar cells. Joule 2022, 6, 816–833. 10.1016/j.joule.2022.03.005.

[ref36] Christians J. A.; Miranda Herrera P. A.; Kamat P. V. Transformation of the excited state and photovoltaic efficiency of CH_3_NH_3_PbI_3_ perovskite upon controlled exposure to humidified air. J. Am. Chem. Soc. 2015, 137, 1530–1538. 10.1021/ja511132a. 25590693

[ref37] Eperon G. E.; Habisreutinger S. N.; Leijtens T.; Bruijnaers B. J.; van Franeker J. J.; deQuilettes D. W.; Pathak S.; Sutton R. J.; Grancini G.; Ginger D. S.; Janssen R. A.; Petrozza A.; Snaith H. J. The importance of moisture in hybrid lead halide perovskite thin film fabrication. ACS Nano 2015, 9, 9380–9393. 10.1021/acsnano.5b03626. 26247197

[ref38] Chen H.; Wei Q.; Saidaminov M. I.; Wang F.; Johnston A.; Hou Y.; Peng Z.; Xu K.; Zhou W.; Liu Z.; Qiao L.; Wang X.; Xu S.; Li J.; Long R.; Ke Y.; Sargent E. H.; Ning Z. Efficient and stable inverted perovskite solar cells incorporating secondary amines. Adv. Mater. 2019, 31, 1903559 10.1002/adma.201903559. 31566819

[ref39] Alsulami A.; Lanzetta L.; Huerta Hernandez L.; Rosas Villalva D.; Sharma A.; Gonzalez Lopez S. P.; Emwas A. H.; Yazmaciyan A.; Laquai F.; Yavuz I.; Baran D. Triiodide formation governs oxidation mechanism of tin-lead perovskite solar cells via a-site choice. J. Am. Chem. Soc. 2024, 146, 22970–22981. 10.1021/jacs.4c01919. 39120593

[ref40] Kresse G.; Furthmüller J. Efficient iterative schemes for ab initio total-energy calculations using a plane-wave basis set. Phys. Rev. B 1996, 54, 11169–11186. 10.1103/PhysRevB.54.11169. 9984901

[ref41] Kresse G.; Joubert D. From ultrasoft pseudopotentials to the projector augmented-wave method. Phys. Rev. B 1999, 59, 1758–1775. 10.1103/PhysRevB.59.1758.

[ref42] Blöchl P. E. Projector augmented-wave method. Phys. Rev. B 1994, 50, 17953–17979. 10.1103/PhysRevB.50.17953. 9976227

[ref43] Perdew J. P.; Burke K.; Ernzerhof M. Generalized gradient approximation made simple. Phys. Rev. Lett. 1996, 77, 3865–3868. 10.1103/PhysRevLett.77.3865. 10062328

[ref44] Grimme S.; Antony J.; Ehrlich S.; Krieg H. A consistent and accurate ab initio parametrization of density functional dispersion correction (DFT-D) for the 94 elements H-Pu. J. Chem. Phys. 2010, 132, 154104 10.1063/1.3382344. 20423165

[ref45] Grimme S.; Ehrlich S.; Goerigk L. Effect of the damping function in dispersion corrected density functional theory. J. Comput. Chem. 2011, 32, 1456–1465. 10.1002/jcc.21759. 21370243

[ref46] Wang D.; Liu Z.-P.; Yang W.-M. Revealing the size effect of platinum cocatalyst for photocatalytic hydrogen evolution on TiO_2_ support: A DFT Study. ACS Catal. 2018, 8, 7270–7278. 10.1021/acscatal.8b01886.

[ref47] Wang D.; Liu Z.-P.; Yang W.-M. Proton-promoted electron transfer in photocatalysis: Key step for photocatalytic hydrogen evolution on metal/titania composites. ACS Catal. 2017, 7, 2744–2752. 10.1021/acscatal.7b00225.

[ref48] Ren G.; Zhou M.; Hu P.; Chen J. F.; Wang H. Bubble-water/catalyst triphase interface microenvironment accelerates photocatalytic OER via optimizing semi-hydrophobic OH radical. Nat. Commun. 2024, 15, 2346 10.1038/s41467-024-46749-z. 38490989 PMC10943107

[ref49] Even J.; Pedesseau L.; Jancu J.-M.; Katan C. Importance of spin–orbit coupling in hybrid organic/inorganic perovskites for photovoltaic applications. J. Phys. Chem. Lett. 2013, 4, 2999–3005. 10.1021/jz401532q.

[ref50] Heyd J.; Scuseria G. E.; Ernzerhof M. Erratum: “Hybrid functionals based on a screened Coulomb potential”. J. Chem. Phys. 2003, 118, 8207 10.1063/1.1564060.

[ref51] Poglitsch A.; Weber D. Dynamic disorder in methylammoniumtrihalogenoplumbates (II) observed by millimeter-wave spectroscopy. J. Chem. Phys. 1987, 87, 6373–6378. 10.1063/1.453467.

[ref52] Weller M. T.; Weber O. J.; Frost J. M.; Walsh A. Cubic perovskite structure of black formamidinium lead iodide, α-[HC(NH_2_)_2_]PbI_3_, at 298 K. J. Phys. Chem. Lett. 2015, 6, 3209–3212. 10.1021/acs.jpclett.5b01432.

[ref53] Baikie T.; Fang Y. N.; Kadro J. M.; Schreyer M.; Wei F. X.; Mhaisalkar S. G.; Graetzel M.; White T. J. Synthesis and crystal chemistry of the hybrid perovskite (CH_3_NH_3_)PbI_3_ for solid-state sensitised solar cell applications. J. Mater. Chem. A 2013, 1, 5628–5641. 10.1039/c3ta10518k.

