# Supplementary material for: Insights into the Dynamic Electron–Hole Separation Process Induced by a Trapped Electron in Lead Halide Perovskites in the Presence of Solutions
Source: JACS Au. 2025 Mar 18;5(4):1738–45. doi: 10.1021/jacsau.4c01261 (PMC12041953; doi:10.1021/jacsau.4c01261)
Supplement: Supplementary file 1 — au4c01261_si_001.pdf [file au4c01261_si_001.pdf]

Supporting Information

Insight into the Dynamic Electron-hole Separation Process Induced by a Trapped Electron in Lead Halide Perovskites in the Presence of Solutions

Yunxuan Ding[a], Yujie Shen[a], Ming-Hsien Lee[b], Haifeng Wang[c]*, P. Hu[a,d]* and Meilan Huang[a]*

[a]School of Chemistry and Chemical Engineering, The Queen's University of Belfast, Belfast, BT9 5AG, UK.
[b]Department of Physics, Tamkang Univeristy, New Taipei 25137, Taiwan
[c]Key Laboratory for Advanced Materials, Centre for Computational Chemistry and Research Institute of Industrial Catalysis, East China University of Science and Technology, Shanghai 200237, China.
[d] School of Physical Science and Technology, ShanghaiTech University, Shanghai, 201210, China.

Email: hupj@shanghaitech.edu.cn (p.hu@qub.ac.uk), hfwang@ecust.edu.cn, m.huang@qub.ac.uk

S1. Electronic property of the $MAPbI_3$ with I dimer

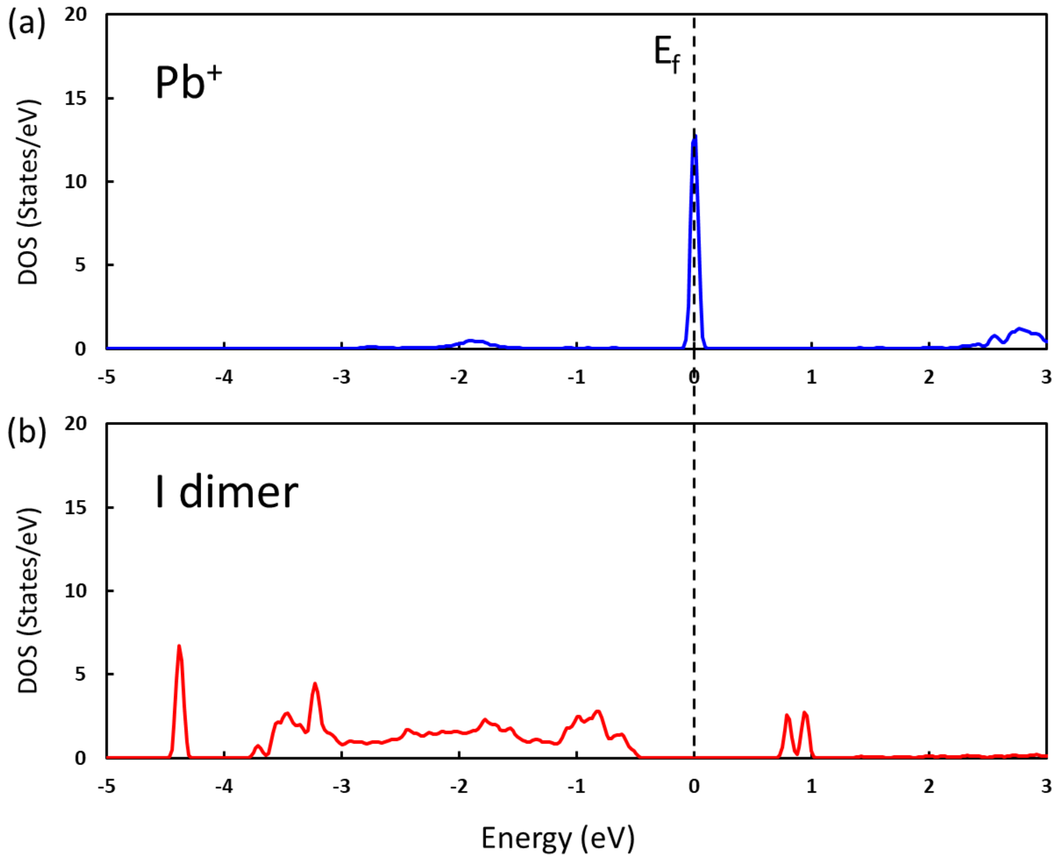

Figure S1. TDOS of (a) the single atom of Pb^+ with trapped electrons and (b) I dimer with trapped holes on the $MAPbI_3$ surface with SOC. The Fermi level is set at 0 eV.

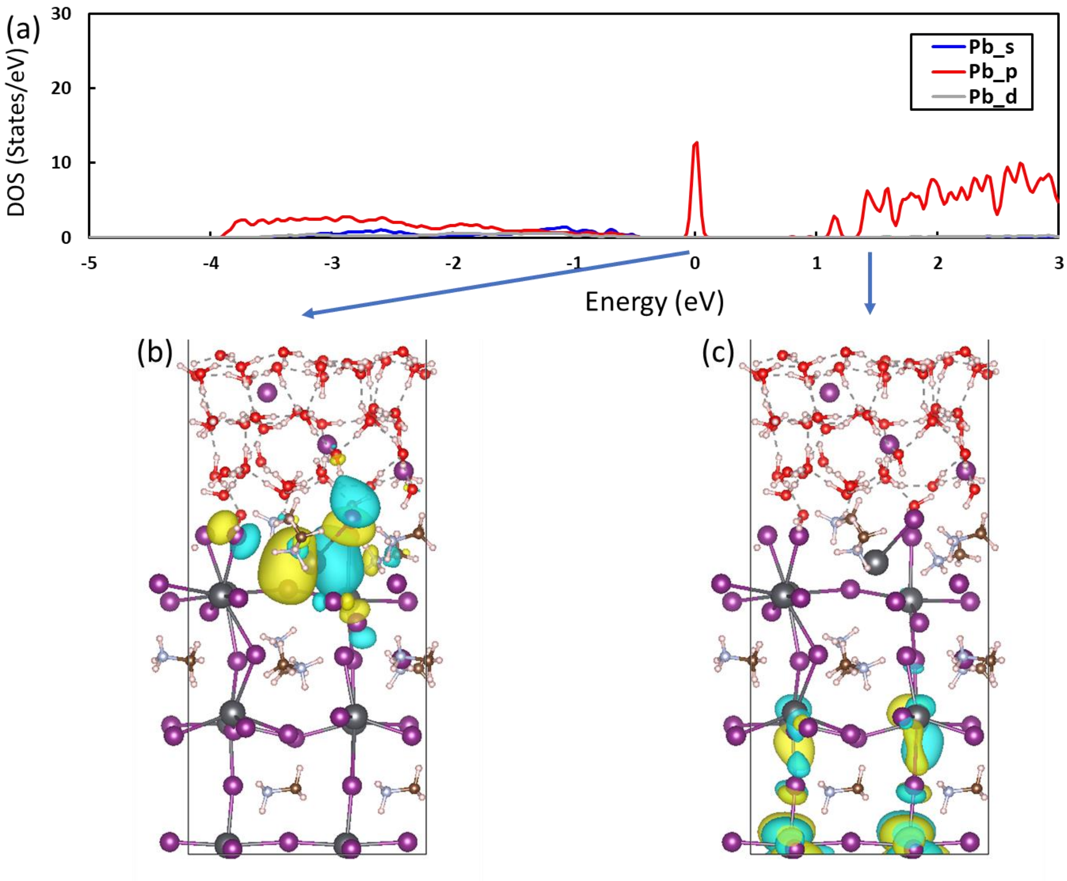

Figure S2. (a) PDOS of Pb in the $MAPbI_3$ surface with SOC. The Fermi level is set at 0 eV. Calculated isosurfaces of wavefunctions in real space for Pb at (b) trapped states and (c) CBM, respectively. The iso-value is 5×10^{-9} e/$Å^3$. The Fermi level is set at 0 eV.

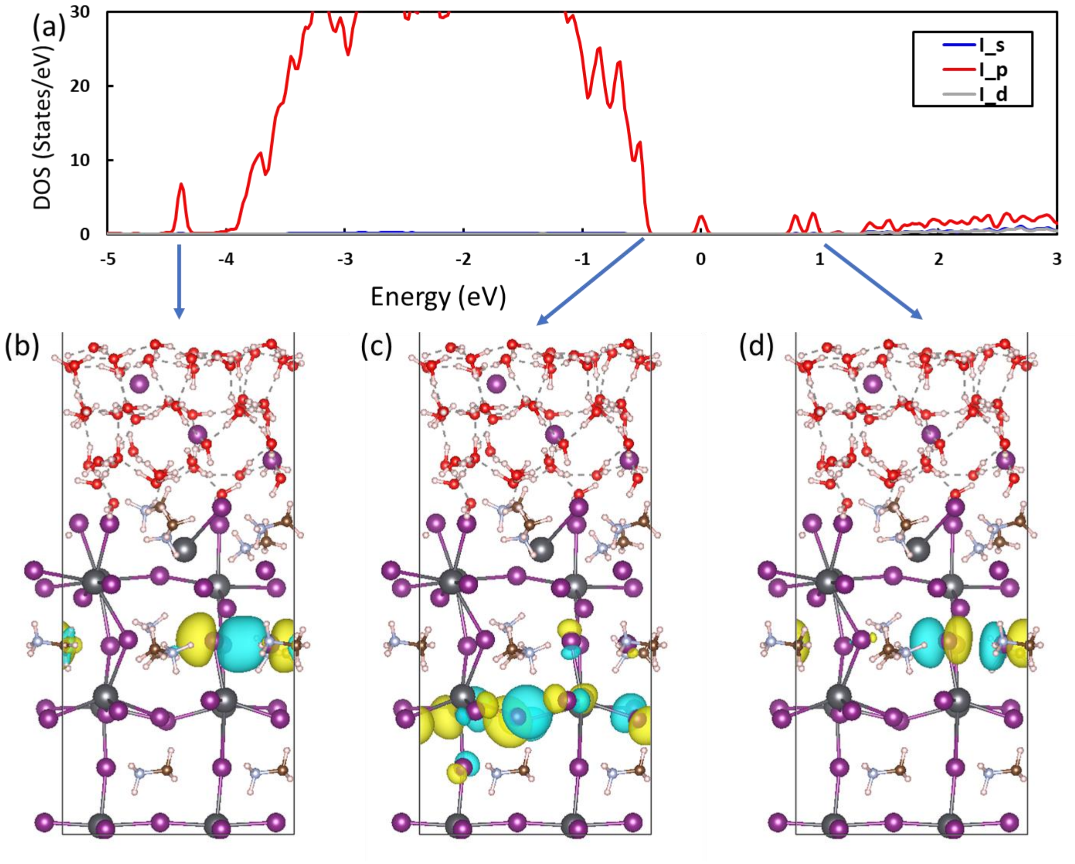

Figure S3. (a) PDOS of I in the $MAPbI_3$ surface with SOC. The Fermi level is set at 0 eV. Calculated isosurfaces of wavefunctions in real space for I at (b) -4.38 eV, (c) VBM, and (d) trapped states, respectively. The iso-value is 5×10^{-9} e/Å^3. The Fermi level is set at 0 eV.

S2. Energy plot of the separated electron and hole recombination

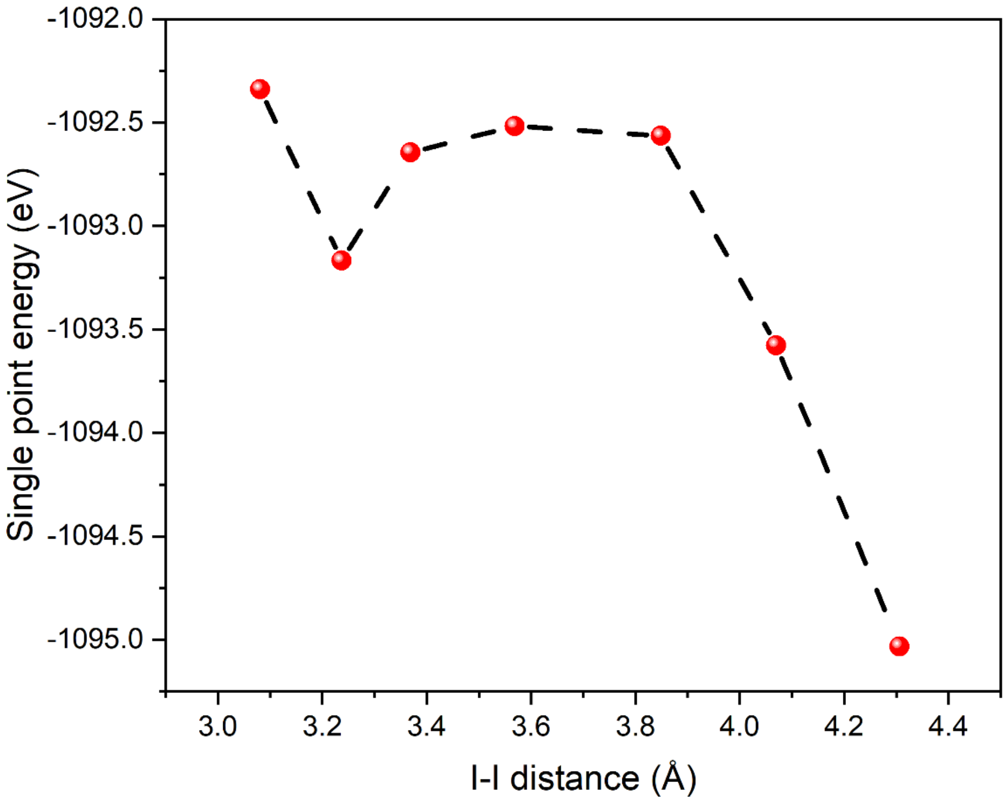

Figure S4. Energy changes as a function of the I dimer distance.

S3. Iodide migration in the I dimer

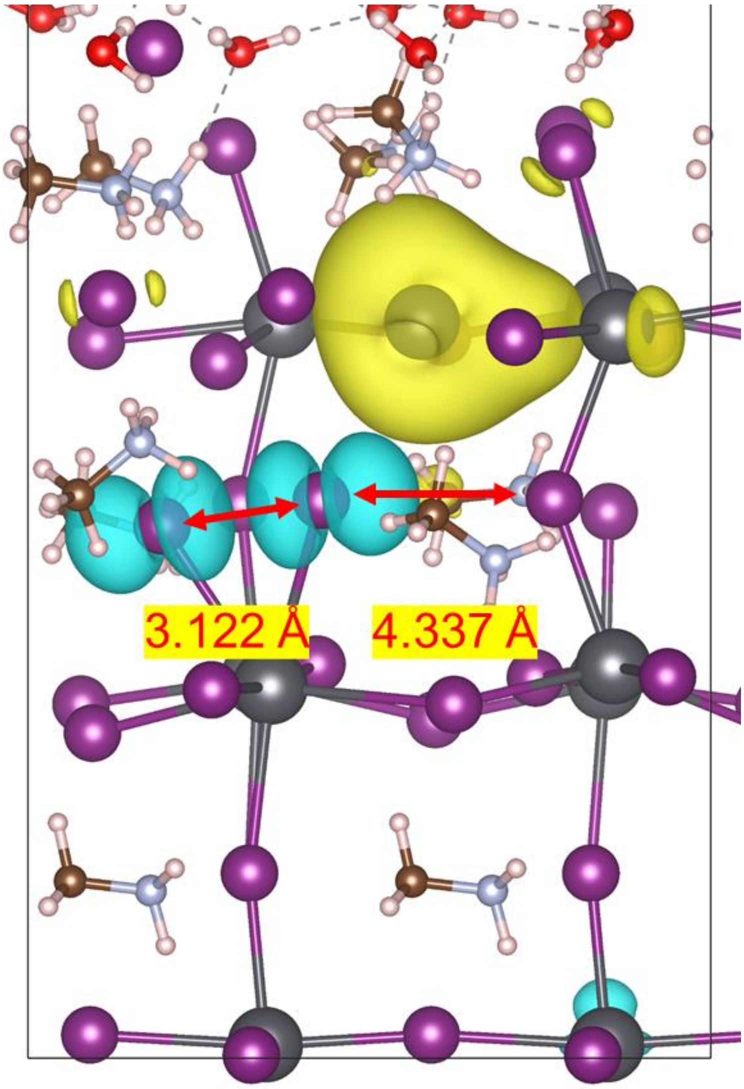

Figure S5. Dynamic geometric structures and spin densities of MAI-terminated $MAPbI_3$ surfaces in 3.15 mol/L HI solutions with an extra electron. The iso-value is 0.002 e/Å^3. The characteristic distance is marked by the line with its value beside. This I dimer is different from the one in Figure 3.

S4. Coexisting state of the trapped electron and hole in the optimized structure

A larger supercell was chosen for the following reason: During the optimization of the original dynamic structure from the MD simulation, the coexisting trap state of an electron and a hole is disappeared with the I dimer separated into two iodides, indicating that it was difficult for Pb^+ to receive more excess electrons separated from the electron-hole pair to generate a Pb^0 atom. The excess electron can only be localized on the other coordinated saturated Pb^{2+}. However, the optimization of the simulated structure shows that a $p(2\times2)$ supercell was too crowded for two Pb^+ on the top layer. Thus, we used a $p(4\times2)$ supercell with one extra electron for the optimization.

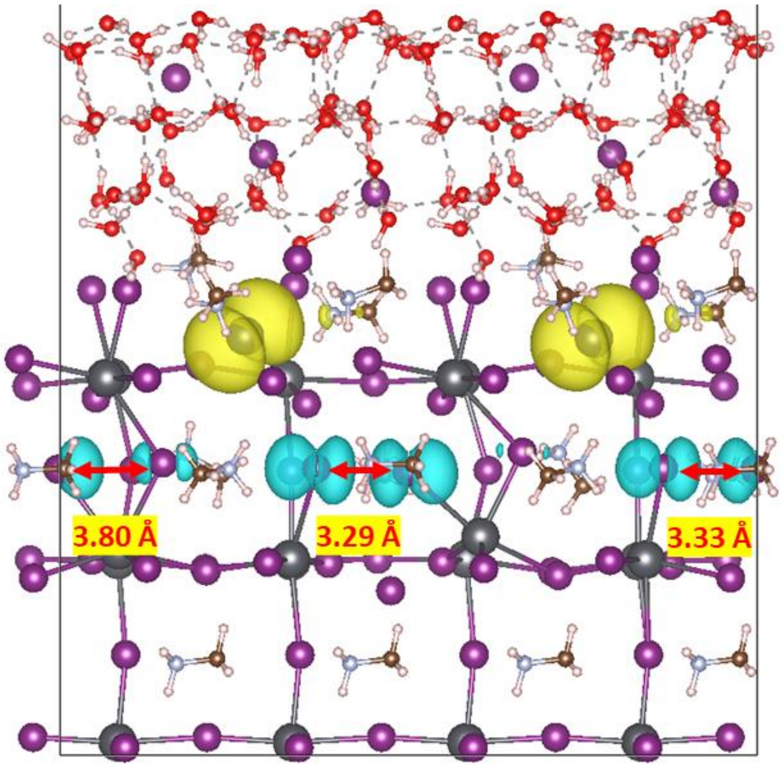

Figure S6. Optimized structure with illustration of spin density for a $p(4\times2)$ supercell $MAPbI_3$ surface in the presence of 3.15 mol/L HI solution with an extra electron. The iso-value is 0.002 e/Å^3.

S5. Delocalized electron on $MAPbI_3$ under dry conditions

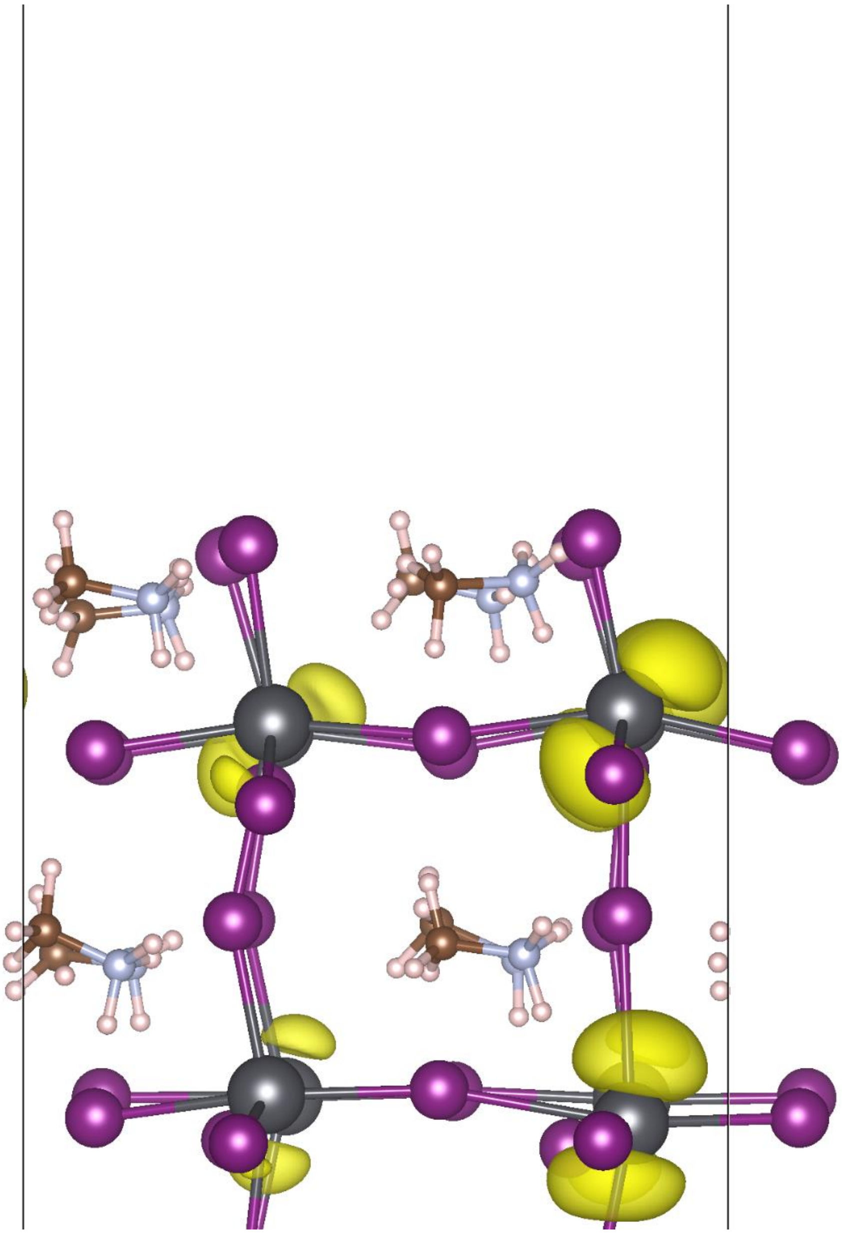

Figure S7. Snapshot with spin densities from MD simulation of MAI-terminated $MAPbI_3$ surfaces with an extra electron under dry conditions. The iso-value of spin densities is 0.0005 e/$Å^3$.

S6. Comparison of DOS calculated by different functionals

To verify the credibility of the DOS analysis employed in this work, calculations were conducted both with and without SOC to study the behavior of electron and hole trapped states. As shown in Figure S6, in comparison to DFT+U, the utilization of SOC did not alter the coexisting state of trapped electrons and holes. Moreover, HSE06 hybrid functional was also used to perform the DOS calculation. Apparently, an occupied polaron peak corresponding to Pb^{+} above the VBM and unoccupied polaron peaks associated with I dimer below the CBM consistently emerged. Moreover, the oxidation states of the Pb ion that traps electrons were compared using the three distinct approaches, which produce +0.11 |e| from DFT+U, +0.15 |e| from HSE06, and +0.13 |e| from SOC, respectively. Although the numbers of charges on the Pb ion from the three different approaches differ slightly, they all show that the electrons are localized on the Pb ion. These findings collectively signify that the incorporation of SOC and the utilization of higher-accuracy functionals do not fundamentally change the intrinsic properties of these localized states.

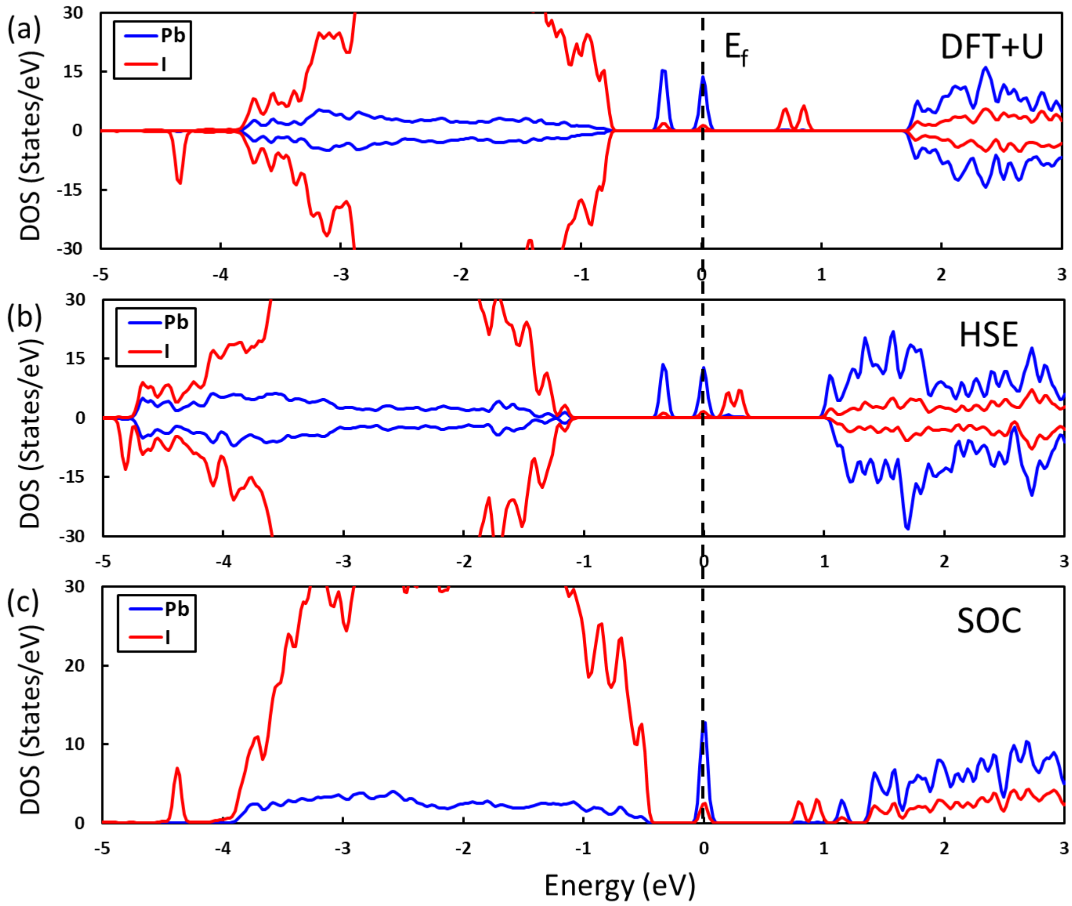

Figure S8. TDOS of the electron-hole coexisting state of Pb and I on the $MAPbI_3$ surface calculated by (a) DFT+U, (b) HSE06, and (c) DFT+U+SOC, respectively. The Fermi level is set at 0 eV.
